# Knowledge of retinopathy of prematurity among pediatricians in King Abdulaziz University hospital in Jeddah: a cross-sectional study

**DOI:** 10.1186/s12886-023-02829-0

**Published:** 2023-03-13

**Authors:** Khadijah Al Attas, Tala Roblah, Salma AlSwealh

**Affiliations:** 1 Department of Ophthalmology, King Abdulaziz University, Jeddah, Saudi Arabia; 2 Faculty of Medicine, King Abdulaziz University, Jeddah, Saudi Arabia

**Keywords:** Retinopathy of prematurity, Premature neonates, Pediatricians

## Abstract

**Background:**

Retinopathy of prematurity (ROP) is a retinal vasoproliferative disorder that affects preterm infants. ROP is a cause of preventable blindness in both developed and developing countries. Pediatricians play a major role in the early detection of ROP, which leads to better overall outcomes for these infants. However, various studies in the literature have reported poor knowledge of the risk factors, prevention, screening, and treatment modalities of ROP among pediatricians. Hence, this study aimed to assess the knowledge and awareness of ROP among pediatricians in Jeddah.

**Methodology:**

This was a cross-sectional study performed among 66 pediatricians at King Abdulaziz University Hospital (KAUH) in Jeddah. A self-administered questionnaire was distributed, and data were collected from March 2022 to October 2022. The questionnaire included sex, level of training, years of practice, and questions that assessed pediatricians’ knowledge of the risk factors for ROP, screening guidelines, referral facilities, and barriers to referral.

**Results:**

Sixty-six pediatricians were included in this study. The cohort showed an equal distribution of males and females (50% each). All of the participants knew that ROP affects the retina (100%). Furthermore, the majority knew that screening should be performed by an ophthalmologist (89.4%), were aware of the risk factors (87.9%), knew that ROP is treatable (90%), and knew that ROP is preventable (70%), and some reported facing obstacles when consulting ophthalmologists (10%). The lack of knowledge was more prevalent among junior residents (56.5%) than among consultants (6%).

**Conclusion:**

This is the first study in the western region of Saudi Arabia to assess the knowledge of ROP among pediatricians. The results showed that a lack of knowledge of screening guidelines and service delivery for ROP exists among pediatricians. Hence, awareness of ROP among pediatricians should be raised since pediatricians play a pivotal role in the early detection of ROP.

## Background

Retinopathy of prematurity (ROP) is a vasoproliferative disorder that affects the retinal vessels [1]. Major risk factors for developing ROP include premature birth at or before 32 weeks, a birthweight of 1500 g or less, poor postnatal growth, and oxygen stress at or soon after birth [1, 2]. ROP is a treatable cause of blindness [3]. A study conducted by Blenocowe H et al. in 2012 reported that 184,700 preterm infants developed any stage of ROP, 10,800 of whom became visually blind from ROP [4]. In Saudi Arabia, the prevalence rate varies between 30 and 56% [4–8]. Prompt screening of ROP among high-risk neonates is vital to provide optimal intervention and prevent blindness. Thus, pediatricians should be well informed about the risk factors and the recommended guidelines for ROP screening [2].

Surprisingly, studies conducted in Palakkad, South India, Thailand, Vietnam, and China stated that the knowledge of ROP was poor among pediatricians [1, 8]. A study conducted in Al Qassim in 2021 showed that half of pediatricians had poor knowledge, and knowledge was substantially lower among those with < 5 years of experience and pediatric residents [9]. Similarly, another study in India stated that only 14.5% of pediatricians followed recommendations for ROP and referral, and their knowledge about screening was poor [1]. In contrast, a study in Nigeria showed that most pediatricians attending a course had sufficient knowledge regarding ROP [1], but they needed to gain knowledge about screening protocols due to the lack of skilled ophthalmologists and inconsistent guidelines [10]. Another survey revealed that pediatricians who screened according to the protocol did not adhere to the Indian guidelines. To illustrate this, only half of the pediatricians considered either the gestational age or birth weight when referring [11].

The lack of awareness is not limited to risk factors and screening but also includes treatment modalities. For instance, 44.6% of pediatricians lacked information about treatment modalities of ROP, and 18.1% thought ROP was not treatable [1]. Since the impact of ROP on neonates is considerable, early diagnosis and intervention through timely screening by pediatricians are pivotal [2].

Saudi Arabia follows the American Academy of ROP screening guidelines and has two programs in the Ministry of Health: the National Eye Health Program and the Neonatology Services Improvement Program. Although there are established ROP screening guidelines, their implementation is suboptimal [12].

The American Academy of Pediatrics, the American Association for Pediatric Ophthalmology and Strabismus, and the American Academy of Ophthalmology, whose guidelines KAUH follows, recommend screening for ROP in all infants with a birth weight less than 1500 g, a gestational age of 32 weeks or less, those with an unstable clinical course such as a requirement for cardiorespiratory support, or those judged to be at high risk by their pediatricians. Screening should be performed by indirect ophthalmoscopy following pupillary dilatation [2, 12–15]. Although several factors influence the frequency and severity of disease development, patients' visual outcomes improve with early treatment with cryotherapy, laser photocoagulation, and anti-vascular endothelial growth factor (VEGF) therapy [16].

The studies mentioned earlier show the importance of the pediatrician in terms of early detection through screening and improvement of the quality of life for patients and their families. To the best of our knowledge, no studies that address the knowledge of ROP among pediatricians have been conducted in Saudi Arabia. Hence, our study aimed to assess the knowledge and awareness of ROP among pediatricians in Jeddah.

## Methods

### Study design and setting

This cross-sectional study was conducted at King Abdulaziz University Hospital (KAUH), Jeddah, Saudi Arabia.

### Sample size and sampling procedure

We analyzed data from 66 pediatricians from all levels (residents, specialists, and consultants) who work in the pediatric department at KAUH in Jeddah; those who refused to participate or had missing data were excluded from this study.

### Data collection

After obtaining consent, the data were collected through email, social media, and clinics from March 2022 to October 2022 in a Google Forms data collection sheet. The following data were gathered: demographic data including age, sex, years of practice, and knowledge about ROP (the effect of ROP in the retina, percentage of ROP among premature neonates, causes of ROP, ROP screening guidelines, treatment options, and the progress of ROP), participants’ practices regarding ROP risk, and whether participants had any barriers in consulting ophthalmologists and the reason.

### Statistical analysis

For knowledge questions (*n* = 10), a score of “1” was given to correct answers, while a score of “0” was given to incorrect answers. The total score and its percentage were computed. Participants who scored 60% or more were considered to have “sufficient knowledge”, whereas those who scored below 60% were considered to have “insufficient knowledge”. Data entry was performed using Microsoft Excel 20, ( Microsoft Corporation,Redmond, Washignton,USA) and statistical analysis was performed using SPSS version 26 (IBM Corporation, Armonk, NY, USA). Descriptive statistics are presented as frequencies and percentages, and univariate analysis was performed using a chi-square test. Multivariate logistic regression analysis was applied to assess the relationships in this study. Odds ratios, 95% confidence intervals and *p* values were generated, with a *p* value < 0.05 considered significant.

### Ethical approval

The biomedical ethics committee of KAUH gave their approval to this study (Reference no. 120/22).

## Results

This study included 66 physicians, and their demographics are summarized in Table 1. They were equally distributed according to sex.

**Table 1:** Demographic characteristics of the participants (*n* = 66)

	**Frequency**	**Percentage**
**Sex**		
Male	33	50.0
Female	33	50.0
**Job title**		
Junior resident (R1-R2)	23	34.8
Senior resident (R3-R4)	16	24.2
Specialist	10	15.2
Consultant	17	25.8
**Years of practice**		
< 5	44	66.7
5–10	6	9.1
> 10	16	24.2

### Knowledge questions about ROP

**Fig. 1:**
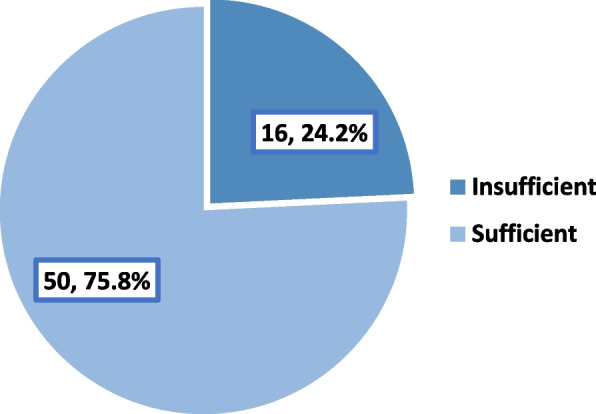
Level of participants’ knowledge about ROP

Table 2 summarizes the participants’ responses to knowledge questions about ROP. All of them could recognize that prematurity affects the retina, and the majority of them (89.4%) knew that an ophthalmologist should perform a screening test for premature neonates. Most of them could recognize the risk factors for ROP (87.9%). Additionally, the majority knew that ROP is preventable (72.7%) and treatable (90.9%). Approximately 60% of them knew that the ideal timing (in chronological age) to start ROP screening depends on the gestational age at birth. Three-quarters (75.8%) expressed sufficient knowledge about ROP, as illustrated in Fig. 1.

**Table 2:** Responses of the participants to knowledge questions about ROP

	**Correct answers**		
	Response	N	%
**What causes retinopathy of prematurity (ROP)?**	Gestational age < 34–35 weeks, weight less than 1500 g and O_2_ requirement for more than 30 days	58	87.9
**What is the ideal timing (in chronological age) to start ROP screening?**	Depends on the gestational age at birth	40	60.6
**What is/are the available options for treating ROP?**	Both laser photocoagulation and intravitreal anti-VEGF injection can be used depending on the case	33	50.0
**Generally, what is the prognosis for ROP?**	Varies depending on early detection, frequent follow-up and intervention	56	84.8

Table 3 summarizes the factors related to the participants’ knowledge about ROP.

**Table 3:** Factors associated with level of participants’ knowledge about ROP: Univariate analysis

	**Knowledge about retinopathy of prematurity**		
Variables	Insufficient	Sufficient	*p* value
	*N* = 16	*N* = 50	
	N (%)	N (%)	
**Sex**			
Male (*n* = 33)	9 (27.3)	24 (72.7)	
Female (*n* = 33)	7 (21.2)	26 (78.8)	0.566
**Job title**			
Junior resident (R1-R2) (*n* = 23)	13 (56.5)	10 (43.5)	
Senior resident (R3-R4) (*n* = 16)	1 (6.3)	15 (93.8)	
Specialist (*n* = 10)	1 (10.0)	8 (90.0)	
Consultant (*n* = 17)	1 (5.9)	16 (94.1)	< 0.001
**Years of practice**			
< 5 (*n* = 44)	15 (34.1)	29 (65.9)	
5–10 (*n* = 6)	0 (0.0)	6 (100)	
> 10 (*n* = 16)	1 (6.3)	15 (93.8)	0.029

Multivariate logistic regression analysis revealed that, compared to junior residents, senior residents, specialists, and consultants were more likely to have sufficient knowledge about ROP (adjusted odds ratios (AORs) and their 95% confidence intervals (CIs) were 19.5 (2.19–173.37), *p* = 0.008; 11.70 (1.27–108.27), *p* = 0.030 and 20.80 (2.35–184.28), *p* = 0.006, respectively). After controlling for confounders, years of clinical experience was not significantly associated with the level of knowledge about ROP.

In terms of participants’ usual practices for ROP risk for a premature baby at the NICU, consulting the ophthalmology department was mentioned by the majority of them (87.9%). Only 10.6% of participants reported facing limitations in reaching an ophthalmologist. The most frequently reported limitation was difficulty contacting an ophthalmologist, even if they were available (7.6%).

## Discussion

ROP is a leading cause of visual disability. Pediatricians, the primary caregivers of premature neonates, should be aware of the risk factors, screening protocol, and treatment options for ROP. Pediatricians’ knowledge will improve the outcomes and quality of life of these neonates [10]. In our study, all participants recognized the etiology of ROP, and the majority knew that an ophthalmologist should perform the screening test for premature neonates. On the other hand, another study conducted in India stated that only 65.1% of pediatricians were aware of the etiology of ROP [3]. A study conducted in Nigeria showed that 95.8% of pediatricians knew the risk factors for ROP. The Proceedings of the World ROP Congress conducted a similar study in Hyderabad [10] and found that 100% of pediatricians were aware of the risk factors. In contrast, Sathiamohanraj et al. showed that only 57.8% of the pediatricians included in their study were aware of the risk factors [3]. This variation in the level of knowledge of the risk factors for ROP should be addressed since ROP is a preventable disease. By identifying risk factors, ROP can be detected early, and detrimental outcomes can be avoided.

There is also variation in terms of awareness of screening protocols. Uhumwangho et al. reported that only 27% of the pediatricians included in their study were aware of these [10]. Likewise, only 60% of the subjects included in our study were aware of the ideal screening timing for ROP. Another study reported that 45.8% of pediatricians did not know the appropriate time to start ROP screening [3]. The lack of clear established screening protocols for ROP in Saudi Arabia might have been the reason behind the lack of awareness of the screening protocols [1].

Unfortunately, 10% of the pediatricians in our study thought that ROP was untreatable. Similarly, Sathiamohanraj reported that 18.1% of pediatricians thought that ROP was untreatable [3]. This could lead to delays in referral, follow-up, and treatment, leading to a worse prognosis. Regarding the pediatrician’s attitudes and practices toward ROP, surprisingly, 12% of the pediatricians did not consult the ophthalmology department when they encountered a premature neonate. Additionally, 10% reported difficulty contacting an ophthalmologist. A similar study reported that the main obstacle faced by pediatricians was the nonavailability of trained ophthalmologists [17].

This study’s strength lies in it being the first to evaluate the awareness of ROP among pediatricians in the western region of Saudi Arabia. Furthermore, we compared differences in knowledge level among consultants, specialists, and residents. Multivariate logistic regression analysis revealed that senior residents, specialists, and consultants were more knowledgeable than junior residents. The results of our study highlight the need to raise awareness about ROP among pediatricians.

Our study had some limitations, such as the small sample size, and it did not include other health care providers, such as NICU nurses. Thus, more studies with a larger and more inclusive sample are needed. Awareness of ROP should be raised among pediatricians, and information should be well disseminated through medical conferences, articles, symposiums, and social media posts.

## Conclusion

In conclusion, the majority of the subjects included in our study showed sufficient knowledge about ROP. Moreover, as expected, consultants, specialists, and senior residents were more likely to have sufficient knowledge than junior residents. To our surprise, few pediatricians reported facing obstacles in reaching an ophthalmologist at their workplace. Hence, awareness of ROP should be raised by focusing on risk factors, screening, prevention, and treatment modalities. This could be achieved in various ways, such as social media, campaigns, lectures, and seminars. The outcomes of ROP can be greatly improved if it is detected early and treated.

## Data Availability

The datasets used and/or analyzed during the current study are available from the corresponding author upon reasonable request.

## Abbreviations

ROP: Retinopathy of prematurity
KAUH: King Abdulaziz University Hospital
AOR: Adjusted odds ratio
CI: Confidence interval
B: Slope
SE: Standard error
